# Two cost-effective techniques for intrarenal pressure monitoring during flexible ureteroscopy: validation of a handheld pressure meter against a sphygmomanometer in a bench-top model

**DOI:** 10.1007/s00345-025-06153-8

**Published:** 2025-12-29

**Authors:** Mohamed Omar, Mohamed F. Sultan, Kemal Sarica, Ioannis Kartalas Goumas, Vineet Guahar, Manoj Monga

**Affiliations:** 1 Urology Department, Menoufia University, Shibin El Kom, Menoufia Egypt; 2 Department of Urology, Medical School, Biruni University, Istanbul, Turkey; 3 Istituto Clinico Beato Matteo, Vigevano, Italy; 4 Department of Urology, Ng Teng Fong General Hospital, Jurong East, Singapore; 5 Urology Department, San Diego School of Medicine, University of California, San Diego, USA

**Keywords:** Intrarenal pressure, Flexible ureteroscopy, Sphygmomanometer, Handheld pressure meter, Cost-effective monitoring

## Abstract

**Background and objective:**

To validate a simple, inexpensive method for intrarenal pressure monitoring during flexible ureteroscopy by comparing a handheld pressure meter with a sphygmomanometer in a bench-top balloon model.

**Methods:**

A balloon was mounted on the distal tip of an 8.4 Fr flexible ureteroscope and connected to a three-way stopcock at the irrigation port. This setup enabled simultaneous pressure recording using a mercury sphygmomanometer (gold standard) and the Wellead handheld pressure meter (Shenzen JCR Medical Technology, China). Thirty paired measurements were obtained across clinically relevant pressure ranges. Agreement was evaluated using paired t-test and Bland–Altman analysis.

**Key findings and limitations:**

Paired t-test showed no significant difference between devices (t = 0.314, *p* = 0.756). The mean bias (Wellead – sphygmomanometer) was 0.02 mmHg with a standard deviation of 0.35 mmHg. The 95% limits of agreement were narrow (−0.664 to +0.704 mmHg). Bland–Altman analysis demonstrated excellent concordance without proportional bias.

**Conclusions and clinical implications:**

This study validates two inexpensive, reproducible methods for intrarenal pressure monitoring via the irrigation port of a flexible ureteroscope: the sphygmomanometer and the Wellead handheld meter. Both demonstrated excellent agreement, offering globally accessible options for laboratory research and potential clinical use.

## Introduction

Intrarenal pressure during flexible ureteroscopy directly influences both procedural safety and patient outcomes. Excessive irrigation increases intrarenal pressure and creates complications including mucosal trauma, forniceal rupture, or infectious sequelae [1].

Currently several novel intrarenal pressure monitoring methods are developed which monitor and help regulate the IRP, integral for FURS in modern endourology [2].

Real-time monitoring with the novel flexible and navigable suction ureteral access sheath (FANS) demonstrated safe intrarenal pressures during flexible ureteroscopy with several clinical advantages, including the vascular Pressure Wire [3] that demonstrated real-time intrarenal pressure monitoring during flexible ureteroscopy. However, this technique requires expensive specialized sensor wire hardware and monitoring systems, limiting its cost-effectiveness and widespread usage [4]. Intrarenal pressure is commonly reported in either centimeters of water (cmH₂O) or millimeters of mercury (mmHg), the two standard units of measurement. Since 1 mmHg ≈ 1.36 cmH₂O, consistent conversion is essential for accurate comparison across studies [5]. 

Modern endourology requires suction to be an integral part of flexible ureteroscopy as its dependent superior outcomes both for stone free rate (SFR) and in minimizing infectious complications attributed to raised IRP [6].

With a cost-conscious approach in endourology surgeons constantly seek to re-invent the wheel as is seen by proposals of considering Reused -disposable scopes for FURS, incorporating reusable devices like the GLITZ system to make any ureteroscope direct in scope suction (DISS) capable [7].

As Most modern flexible ureteroscopes have working channels of ~ 3.6 Fr, which are sufficiently wide for pressure monitoring an accurate, inexpensive, and reliable measurement of IRP may be done via the irrigation port of a flexible ureteroscope in both experimental and clinical settings, a simple cost-effective and practical solution to monitor IRP using any commercial ureteroscope.

Traditionally, sphygmomanometers) have been used as simple, low-cost laboratory devices for direct blood pressure measurement. However, their use in experimental or clinical workflows is being replaced by modern more practical battery-operated handheld meters, such as the Wellead, handheld pressure meter (Shenzen JCR Medical Technology, Guangdong, China), these are compact, portable, durable, cheap and facilitate routine real time pressure monitoring during intrarenal surgery.

Our primary aim of this invitro study is to integrate these into the working channel of a 3.6 fr flexible ureteroscope and compare balloon pressure readings obtained with a handheld meter against a sphygmomanometer (gold standard) using a bench-top model designed to simulate flexible ureteroscopy.

## Methods

### Experimental setup

**Fig. 1:**
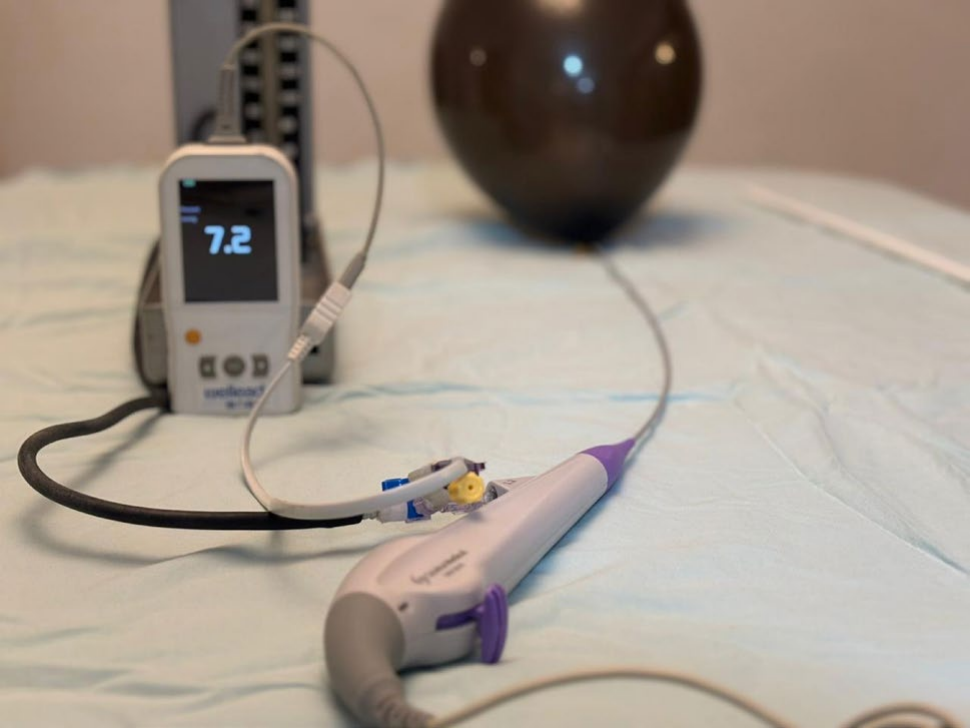
An inflated standard balloon mounted around the distal tip of an 8.4 Fr flexible ureteroscope (Scivita Medical, China) and secured in place using an elastic band

**Fig. 2:**
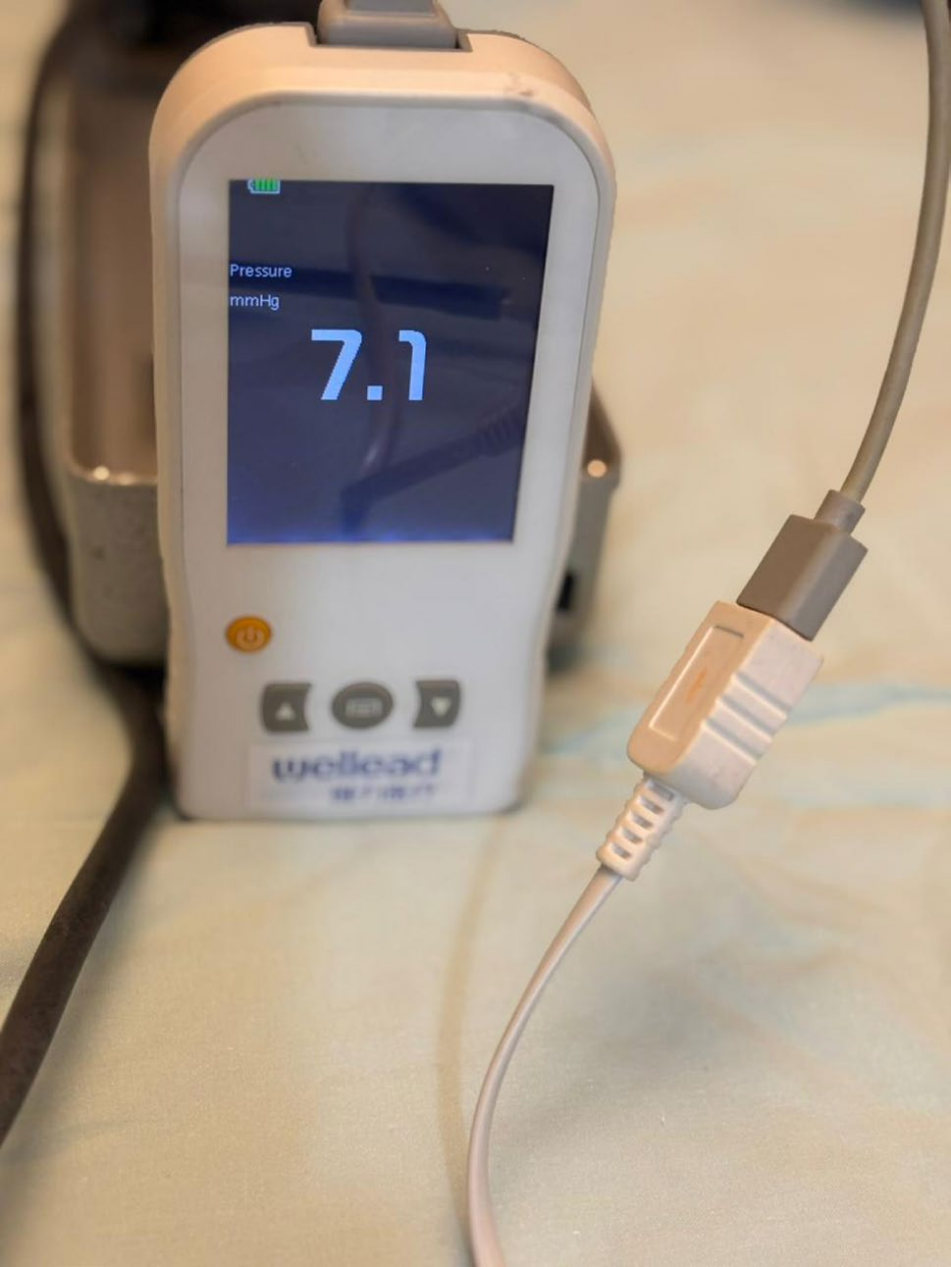
The Wellead handheld pressure meter (Shenzhen JCR Medical Technology, Guangdong, China)

A bench-top model was developed to simulate pressure monitoring during flexible ureteroscopy. A standard balloon was mounted around the distal tip of an 8.4 Fr flexible ureteroscope (Scivita Medical China) and secured in place using an elastic band (Fig. 1). The ureteroscope’s irrigation port (3.6 Fr channel) was connected to a three-way stopcock, permitting simultaneous connection of two pressure measurement devices: the Wellead handheld pressure meter (Shenzen JCR Medical Technology, Guangdong, China) (Fig. 2) and a mercury sphygmomanometer without cuff, which served as the gold standard reference.

### Measurement protocol

**Fig. 3:**
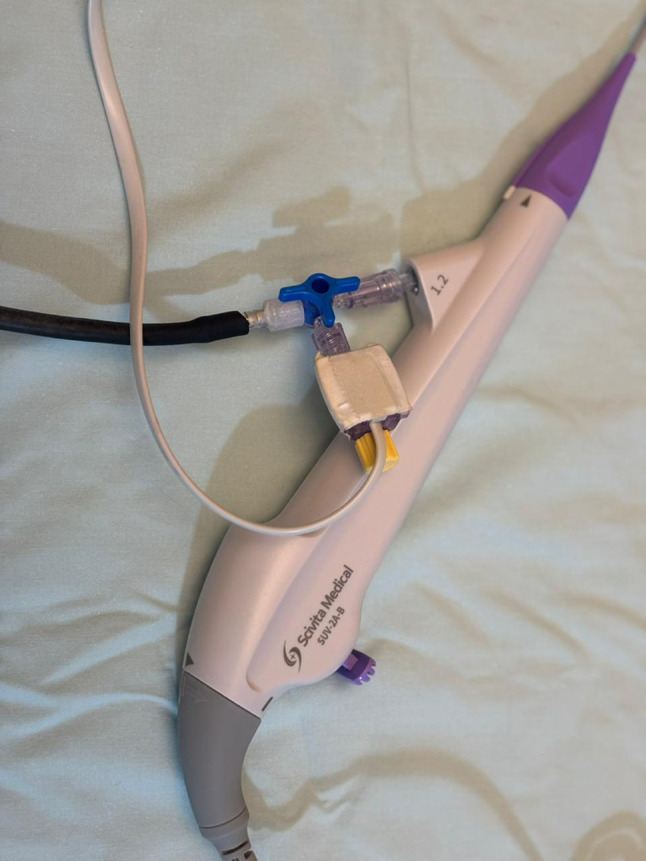
Pressure-monitoring setup with a 3-way connector enabling simultaneous balloon pressure recording by both the sphygmomanometer and the test device

The balloon was progressively inflated using a syringe until clinically relevant pressure levels were achieved. At each inflation step, balloon pressure was recorded simultaneously from both devices via the irrigation channel across the 3-way connector (Fig. 3). This process was repeated across multiple inflations to ensure reproducibility.

### Data collection

A total of 30 paired pressure measurements were obtained. Each pair represented the pressure reading from the Wellead meter and the sphygmomanometer taken under identical balloon inflation conditions. Care was taken to stabilize the system before each recording to avoid fluctuations related to transient irrigation or balloon instability.

### Statistical analysis

Data was analyzed using SPSS version 26 (IBM Corp., Armonk, NY, USA). Continuous variables were expressed as mean ± standard deviation (SD). Agreement between the two devices was assessed using a paired t-test to detect systematic differences, and by constructing a Bland–Altman plot to evaluate mean bias and 95% limits of agreement. Proportional bias was assessed visually. A p value < 0.05 was considered statistically significant.

## Results

**Fig. 4:**
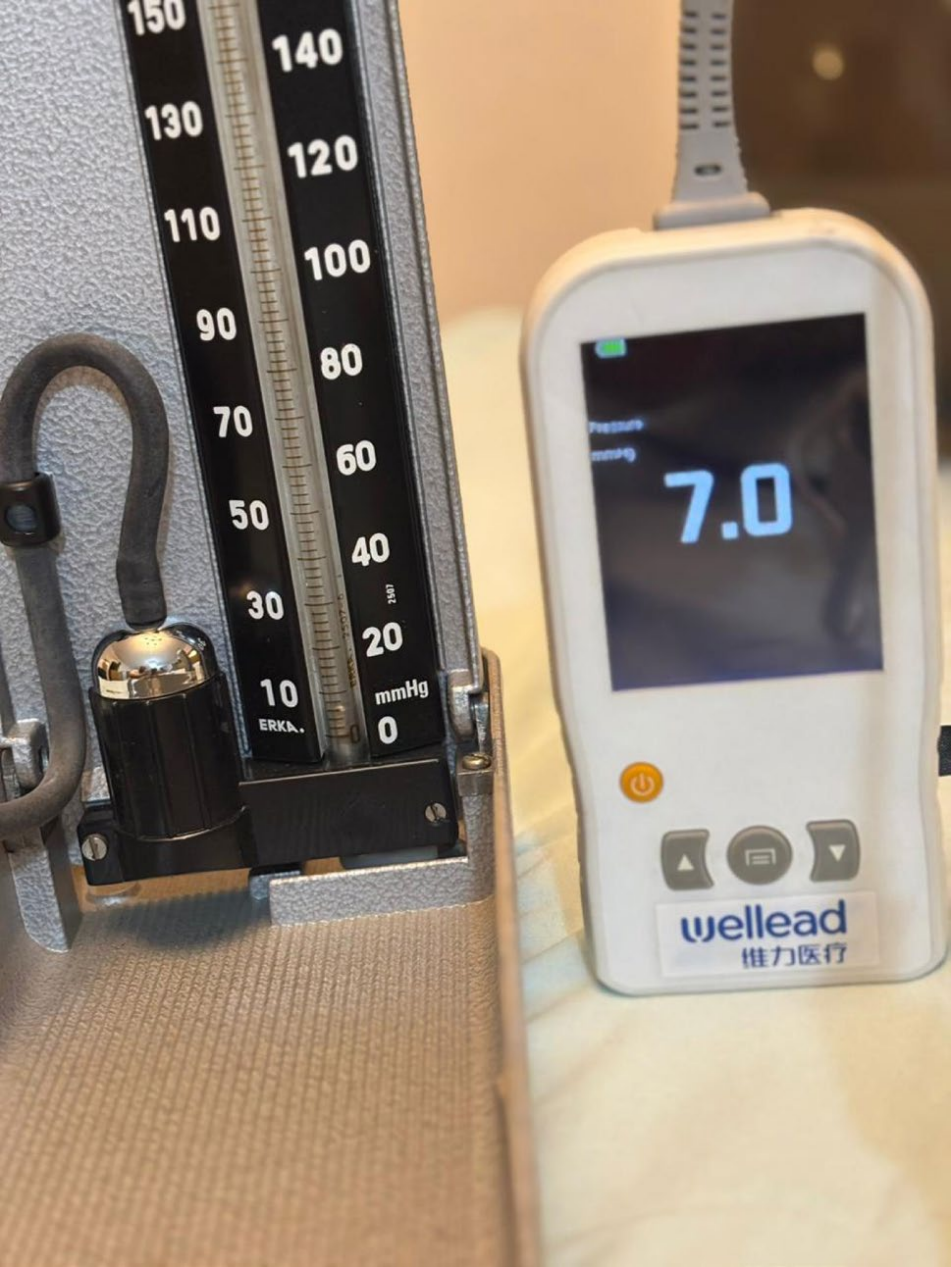
Comparative balloon pressure measurements across both devices

**Fig. 5:**
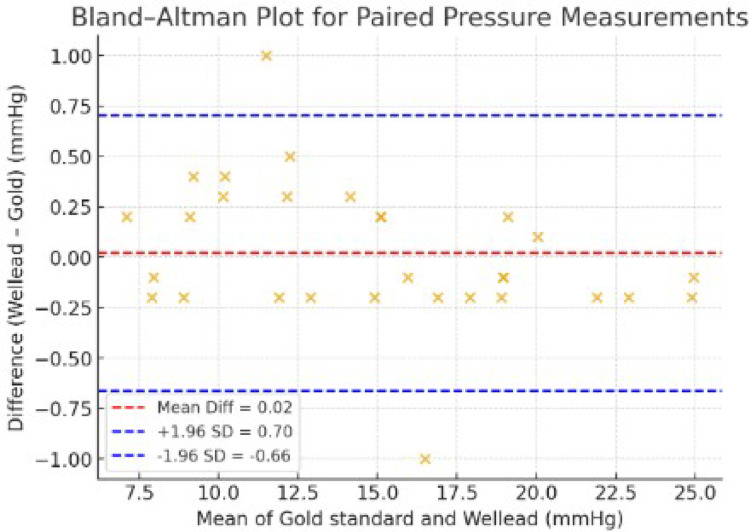
Bland–Altman plot comparing intraluminal pressure measurements obtained with the Wellead handheld pressure meter and the sphygmomanometer (gold standard). The central dashed red line indicates the mean difference (0.02 mmHg). The dashed blue lines represent the 95% limits of agreement (–0.66 to +0.70 mmHg)

A total of 30 paired balloon pressure measurements were analyzed. Paired t-test analysis demonstrated no statistically significant difference between the two devices (t = 0.314, *p* = 0.756) (Fig. 4). The mean bias (Wellead—gold standard) was 0.02 mmHg, with a standard deviation of 0.35 mmHg. The 95% limits of agreement ranged from −0.664 to +0.704 mmHg. The Bland–Altman plot (Fig. 5) illustrates these narrow limits of agreement and shows no evidence of proportional bias (Table 1).

**Table 1:** Paired pressure measurements using sphygmomanometer (gold standard) and Wellead handheld pressure meter (*n* = 30)

Sample ID	Gold standard (mmHg)	Wellead (mmHg)	Difference (Wellead – Gold)	Mean (mmHg)
1	17	16.0	−1.0	16.5
2	19	19.2	0.2	19.1
3	11	12.0	1.0	11.5
4	12	12.5	0.5	12.3
5	19	18.9	−0.1	19.0
6	25	24.8	−0.2	24.9
7	12	12.3	0.3	12.2
8	14	14.3	0.3	14.2
9	9	9.2	0.2	9.1
10	10	10.4	0.4	10.2
11	20	20.1	0.1	20.1
12	15	15.2	0.2	15.1
13	10	10.3	0.3	10.2
14	15	15.2	0.2	15.1
15	7	7.2	0.2	7.1
16	19	18.8	−0.2	18.9
17	18	17.8	−0.2	17.9
18	16	15.9	−0.1	16.0
19	8	7.9	−0.1	7.95
20	15	14.8	−0.2	14.9
21	12	11.8	−0.2	11.9
22	23	22.8	−0.2	22.9
23	13	12.8	−0.2	12.9
24	17	16.8	−0.2	16.9
25	9	8.8	−0.2	8.9
26	22	21.8	−0.2	21.9
27	8	7.8	−0.2	7.9
28	25	24.9	−0.1	25.0
29	19	18.9	−0.1	19.0
30	9	9.4	0.4	9.2

## Discussion

Typically monitoring IRP via scopes requires pressure-sensing guidewire integrated into a scope working channel which measures the pressure inside the kidney via scope tip. This sensor feeds data to a pressure monitoring feedback device, displaying real-time pressure readings on a monitor for the surgeon. The pressure is directly affected by irrigation inflow and outflow, and by factors like scope manipulation and the ureter’s anatomy, with elevated pressures. These scopes are typically single use and more expensive conventional ureteroscopes [8].

This laboratory bench study demonstrates that real-time balloon pressure measurement via the irrigation port of a flexible ureteroscope is both feasible and reliable. The Wellead handheld pressure meter provided virtually identical readings to a sphygmomanometer, the gold standard reference, with negligible bias and very narrow limits of agreement. These findings support the use of handheld devices for accurate intraluminal pressure monitoring during experimental and potentially clinical ureteroscopy Via an empty working channel port.

.

The key innovation in this study lies in the simple yet effective use of a three-way stopcock connected to the irrigation port of a flexibleureteroscope, enabling simultaneous measurement with two devices. This design circumvents the need for expensive sensor wires or integrated pressure systems, providing a low-cost, universally applicable solution for both laboratory and clinical settings. By leveraging the existing irrigation channel (~3.6 Fr in most modern flexible ureteroscopes), this method ensures a stable and unobstructed pathway for pressure transmission, minimizing the risk of blockage by stone dust or irrigation debris in comparison to smaller caliber pressure-monitoring catheters or accessory wires.

In real-life clinical practice, we connect the three-way stopcock to a Y-adapter, which preserves the primary working channel for the laser fiber or Dormia basket. This configuration allows irrigation and pressure measurement to be performed simultaneously through the three-way stopcock without interfering with accessory use, thereby ensuring full procedural functionality while enabling reliable intrarenal pressure assessment.

Compared with costly technologies such as the vascular pressure wire or suction-based ureteral access sheaths, which require costly hardware and complex setups [9], this approach offers a practical alternative. While specialized systems may provide continuous intrarenal pressure monitoring with additional functional benefits, their cost-effectiveness and accessibility remain major barriers to widespread adoption [2]. In contrast, a sphygmomanometer or a portable handheld meter—when coupled with the three-way connector technique—represents a first-time demonstration of an inexpensive, scalable, and reproducible pressure monitoring method.

The strengths of this study include the direct head-to-head comparison with a gold-standard device, the controlled bench model minimizing confounders, and the use of robust agreement analysis via Bland–Altman methodology. Together, these features reinforce the reliability of the results and the validity of the experimental design.

Nonetheless, several limitations must be acknowledged. This was a bench-top model, and clinical variables such as tissue compliance, ureteral peristalsis, urine outflow, and irrigation variability were not accounted for.

A key limitation of the current technique is the need to briefly pause irrigation to obtain a stable pressure reading. Continuous, simultaneous irrigation and pressure measurement are not yet feasible with the present setup, and future refinements should focus on enabling real-time pressure monitoring without interrupting irrigation flow.

Additionally, pressure measurements were limited to balloon inflation through the irrigation channel; future studies should evaluate this method directly in vivo for intrarenal pressure monitoring during actual ureteroscopy. Additionally, this experiment was done with an empty ureteroscopy channel and emulating the same with instruments in place along with irrigation fluid may not be technically feasible unless this is tested in a double lumen scope. Its commercial application will require a modified stopcock mechanism that can be ergonomically challenging, yet our experiment represents a novel minimalistic approach for scope manufacturers to consider. This could potentially reduce the need for costly IRP monitoring systems or specialized monitors for same.

Finally, while the technique is simple and low-cost, its translation into routine surgical practice will require further validation of sterility, integration with irrigation systems, and ergonomic workflow considerations.

This study introduces and validates a novel, cost-effective, and reproducible method for real-time pressure monitoring during flexible ureteroscopy using the irrigation port and a three-way connector. Both the traditional sphygmomanometer and the handheld Wellead meter demonstrated excellent agreement, with the handheld device offering additional portability and ease of use. This concept has the potential to bridge the gap between sophisticated high-cost pressure monitoring technologies and practical, everyday clinical tools.

## Conclusion

This study compares two practical and inexpensive methods for intrarenal pressure measurement via the irrigation port of a flexible ureteroscope: the traditional sphygmomanometer and the Wellead handheld pressure meter. Both techniques are reliable, and reproducible options for real-time pressure monitoring.

## Data Availability

Datasets are available on request.
